# Necrotising epididymo-orchitis with scrotal abscess

**DOI:** 10.2349/biij.1.2.e11

**Published:** 2005-10-01

**Authors:** M Muttarak, W Na Chiangmai, P Kitirattrakarn

**Affiliations:** Departments of Radiology and Surgery, Chiang Mai University, Chiang Mai, Thailand

## HISTORY

A 55-year-old man presented with a painful left scrotal enlargement and fever for 6 weeks. He was treated with antibiotics at the community hospital. His fever and scrotal pain were improved but the scrotum became enlarged. He was referred to our hospital to have testicular tumour ruled out. He had history of left renal calculi and urethral stricture with off and on urinary tract infection. Physical examination revealed left scrotal erythema with an enlarged, tender left scrotum. Laboratory investigations were notable for mild elevation of blood urea nitrogen 36 mg/dL (normal 7-24 mg/dL), and creatinine 1.7 mg/dL (normal 0.6-1.6 mg/dL). Urinalysis revealed cloudy urine with numerous white blood cells and red blood cells. Blood leucocyte count was 6.9 × 10^3^ per dL with 59.6% neutrophils, 31.8% lymphocytes, and 3.5% monocytes. Urine culture showed no growth of organism. Scrotal ultrasonography (US) was performed. The patient underwent left orchiectomy. His post-operative period was uneventful. One month later, left nephrolithotomy was performed.

## IMAGING FINDINGS

**Figure 1:**
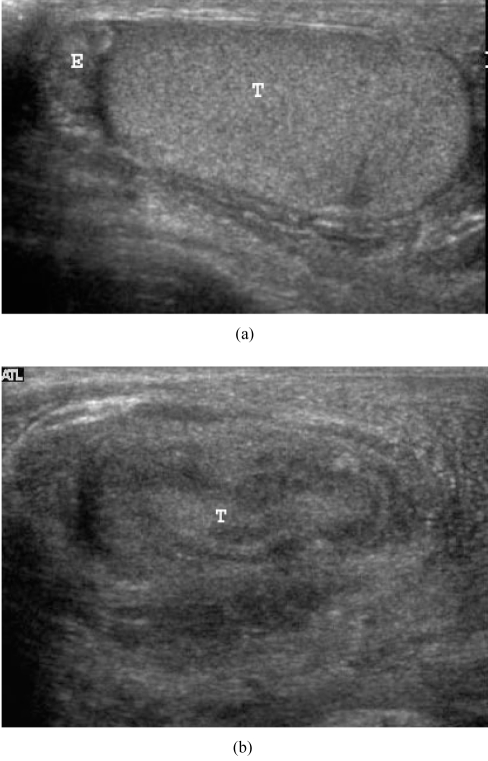
(a) Longitudinal US scan of the right hemiscrotum shows a normal homogeneous echoic epididymis (E) and testis (T). The testicular contour is well defined; (b) Longitudinal US scan of the left hemiscrotum shows an inhomogeneous small testis (T) with loss of normal well-defined contour. The scrotal skin is thickened on the left side compared to the right side.

**Figure 2:**
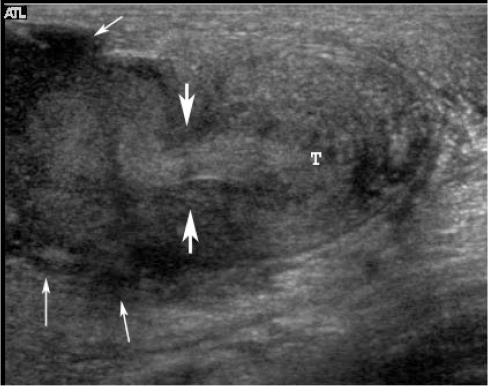
Transverse US scan of the left hemiscrotum shows an inhomogeneous small testis (T), an inhomogeneous echoic tract (thick arrows) connecting to the scrotal abscess (thin arrows), which is seen as a complex echoic mass.

**Figure 3:**
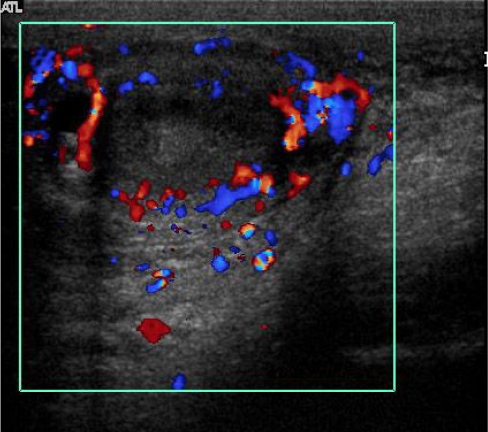
Colour Doppler US of the left testis shows increased peritesticular vascularity but absence of intravascularity indicating testicular ischemia.

Scrotal US showed a normal right testis and epididymis (Figure 1a). The left testis was small with inhomogeneous echogenicity and loss of normal surrounding contour (Figure 1b). The left scrotal skin was thickened. A complex echoic mass in the left scrotal sac with connection to the left testis (Figure 2) was demonstrated, compatible with scrotal abscess. Colour Doppler US at the left hemiscrotum showed increased peritesticular flow but no intratesticular flow (Figure 3).

## SURGICAL FINDINGS

**Figure 4:**
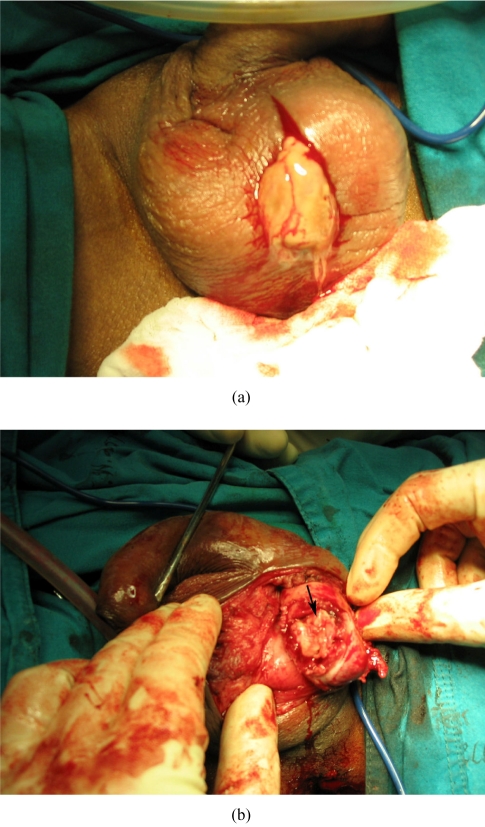
At operation, (a) thick pus was found in the left scrotum; and (b) the left testis was necrotic (arrow).

At operation, the scrotal skin was thickened and inflamed. There was thick pus in the left scrotal sac (Figure 4). The left testis and epididymis were necrotic (Figure 5). Left orchiectomy was performed.

## DISCUSSION

Epididymitis and epididymo-orchitis are two most common causes of acute scrotal pain in adults. The infection usually originates in the genitourinary tract, particularly the bladder, urethra, and prostate. The most common pathogens are *Neisseria gonorrhoea*, *Chlamydia trachomatis*, *Escherichia coli*, or *Proteus mirabilis*. The inflammation usually starts in the epididymis and then spreads to the testis [1-3]. If the patients do not receive appropriate treatment it could result in many complications including pyocoele, testicular infarction, testicular abscess, scrotal abscess, and fulminant fasciitis (Fournier’s gangrene) [1].

Patients with epididymo-orchitis usually present with fever, dysuria, and a painful scrotal enlargement. The pain is usually insidious in onset and increases slowly over 1 to several days. Physical examination may not be possible to differentiate the epididymis and testis due to pain and swelling, making it difficult to evaluate the real extent of the lesion. Differential diagnosis with acute testicular torsion can sometimes be a problem because of similar clinical presentation. US with colour Doppler is helpful in evaluating these patients to prevent unnecessary surgical exploration [1-4]. Grayscale US findings of epididymo-orchits are enlarged hypoechoic epididymis and testis. These findings are non-specific and indistinguishable from testicular torsion but colour Doppler US findings are different. Vascularity is increased in epididymo-orchits but decreased in testicular torsion. However, advanced epididymo-orchitis may cause testicular infarction as a result of extrinsic compression of testicular vascular supply by enlarged epididymis and spermatic cord and pyocoele [1,4-6], therefore, intratesticular vascularity is decreased. This finding suggests the need for surgical intervention. In addition, severe epididymo-orchitis may also cause testicular abscess and scrotal abscess, which is demonstrated as a complex echoic mass.

The presented case shows a severe epididymo-orchitis spreading from a urinary tract infection complicated with testicular necrosis and scrotal abscess.
